# Efficacy of a “Checklist” Intervention Bundle on the Clinical Outcome of Patients with *Candida* Bloodstream Infections: A Quasi-Experimental Pre-Post Study

**DOI:** 10.1007/s40121-020-00281-x

**Published:** 2020-02-04

**Authors:** Antonio Vena, Emilio Bouza, Rafael Corisco, Marina Machado, Maricela Valerio, Carlos Sánchez, Patricia Muñoz, for the COMIC Study Group

**Affiliations:** 1 Clinical Microbiology and Infectious Disease Division, Hospital General Universitario Gregorio Marañón, Madrid, Spain; 2 Instituto de Investigación Sanitaria Hospital Gregorio Marañón, Madrid, Spain; 3 Department of Health Sciences, Infectious Disease Clinic, University of Genoa and Hospital Policlinico San Martino-IRCCS, Genoa, Italy; 4 Medicine Department, School of Medicine, Universidad Complutense de Madrid, Madrid, Spain; 5 CIBER Enfermedades Respiratorias-CIBERES, CB06/06/0058, Madrid, Spain

**Keywords:** Antifungal stewardship, Bundle, *Candida*, Candidemia, Outcome, Quality of care

## Abstract

**Introduction:**

To evaluate the clinical impact of a comprehensive care bundle for the management of candidemia.

**Methods:**

A quasi-experimental pre-post study was implemented. During the pre-intervention period (May 2014–September 2015), a non-mandatory antifungal stewardship program (ASP) was implemented, and patients with candidemia were visited by an infectious disease specialist who provided diagnostic and therapeutic advice according to standard of care as soon as possible. During the post-intervention period (October 2015–May 2017), patients were managed according to a candidemia care bundle with clear and structured recommendations written in their medical history.

**Results:**

Overall, 109 patients were included, 56 in the pre-intervention and 53 in the post-intervention period. Overall, compliance with the *Candida* bundle significantly improved between the pre- [27/56 (48.2%)] and post-intervention [43/53 (81.1%); *p* = 0.01] period. Individual bundle components that significantly improved in the post-intervention period were early adequate antifungal therapy [47/56 (83.9%) vs. 51/53 (96.2%), *p* = 0.05], early adequate source control of the infection [37/56 (82.2%) vs. 41/53 (97.6%), *p* = 0.03] and appropriate duration of therapy [27/56 (48.2%) vs. 43/53 (81.1%), *p* = 0.01]. Adherence to follow-up blood cultures, ophthalmologic examination and echocardiography improved in the post-intervention period, but the difference was not statistically significant. Multivariate analysis revealed that being managed according to candidemia bundle had a favorable impact on 14-day mortality (HR 0.08, 95% CI 0.01–0.45, *p* = 0.02) and 30-day mortality (HR 0.40, 95% CI 0.18–0.89, *p* = 0.02).

**Conclusions:**

A simple bundle focused on increasing adherence to a few evidence-based interventions contributed to a significant reduction in 14- and 30-day mortality in patients with candidemia.

## Key Summary Points

**Table:** 

*Candida* bloodstream infection (BSI) is a life-threating disease associated with significant morbidity, mortality and high healthcare costs.
Prompt diagnosis, early administration of appropriate therapy and adequate source control of infection have been shown to improve the prognosis.
Our hypothesis was that the systematic implementation of structured recommendations aimed at enhancing adherence to evidence-based indicators could improve both diagnostic procedures and antifungal therapy and eventually the outcome of patients with candidemia.
A simple bundle focused on increasing adherence to a few evidence-based interventions contributed to a significant reduction in 14- and 30-day mortality in patients with candidemia.

## Introduction

*Candida* bloodstream infection (BSI) is a life-threating disease associated with significant morbidity, mortality and high healthcare costs. Complications are frequent [1, 2], and mortality ranges between 20 and 45% [3–5].

Prompt diagnosis, early administration of appropriate antifungal therapy and adequate source control of infection have been shown to be key factors that improve the prognosis of patients with candidemia [6–10]. Infectious disease (ID) consultations providing diagnostic and therapeutic bedside recommendations as a part of antifungal stewardship programs (ASP) have also been demonstrated to be associated with a better prognosis for candidemic patients [11–13].

However, the recommendations provided by the ID consultants in such studies were not structured according to a bundle approach, and specific interventions were not always completed in time.

Our hypothesis was that the systematic implementation of structured recommendations aimed at enhancing adherence to evidence-based indicators could improve both diagnostic procedures and antifungal therapy and eventually the outcome of patients with candidemia. Therefore, the aim of this prospective study was to evaluate the all-cause 14- and 30-day mortality in candidemic patients before and after the implementation of a bedside checklist care bundle in our antifungal stewardship program.

## Methods

### Study Setting

This study was performed at the Hospital Universitario General Gregorio Marañón, a 1550-bed tertiary care institution in Madrid, with a full range of clinical services attending a population of approximately 715,000 inhabitants.

### Study Population

All consecutive adult patients (> 18 years of age) with at least one episode of candidemia diagnosed over the May 2014 to May 2017 period were eligible for the study. Exclusion criteria were: patients with a life expectancy of < 72 h or those who were transferred to another healthcare facility.

### Study Design

We performed a pre-post quasi-experimental study. During the pre-intervention period (May 2014–September 2015), a non-mandatory ASP was implemented at our hospital [12], and patients with candidemia were visited by an ID specialist who provided diagnostic and therapeutic advice according to standard of care as soon as possible.

During the post-intervention period (October 2015–May 2017), patients were managed according to a candidemia care bundle. Briefly, two ID physicians with specific expertise in mycology and antifungal therapy followed all candidemic patients and actively promoted adherence to bundle endpoints providing clear and structured recommendations that were written in the patients' medical history. In particular, ID physicians performed follow-up visits of all candidemic patients to (1) review antifungal therapy and change it according to culture results and (2) request (based on the checklist) follow-up blood cultures, ophthalmologic examination or echocardiography. In addition to visits scheduled according to the study protocol (Table 1), further clinical assessments were also performed according to patients' clinical need. Patients were followed up until 30 days from *Candida* BSI onset or until death.

**Table 1:** Candidemia bundle checklist

Day 0	Checklist
Check for sepsis and septic shock	
Presence of ocular symptoms	
Presence of cardiac murmur or intravascular device	
Previous azole use	
Drug-drug interaction	
Reviewing the previous microbiologic cultures	
Choose the adequate antifungal drug according to clinical condition and previous cultures	
Check for adequate antifungal dosage according to weight, renal and hepatic function	
Request all necessary microbiologic and radiologic tests	
Check for the number of CVC and peripheral catheters, as their status. Support all device withdrawal when unnecessary	
If necessary, CVC withdrawal and adequate control of other sources	
Day +1	
Microbiologic adjustment according to E-test and MALDI-TOF results	
Performance of follow-up blood cultures	
Request echocardiography	
Request ophalmoscopy	
Request central venous echography if a clinical suspicion of thrombophlebitis is present	
Day +3	
Check for definitive antifungal susceptibility testing	
Check if antifungal serum concentration is adequate, if clinically necessary	
Check for negativity of previous follow-up blood cultures. If positive, request new blood culture sets	
Check for results of all previous microbiologic cultures	
Check for adequate source control of the infections	
Day +5	
Check for toxicity, drug-drug interactions and renal and hepatic functions	
Check for negativity of previous follow-up blood cultures. If positive, request new blood culture sets	
If possible, step-down therapy	
Day +7	
Check for ophthalmoscopy and echocardiography results	
Check for negativity of previous follow-up blood cultures. If positive, request new blood culture sets	
Day +14	
Check for all microbiologic cultures, ophthalmoscopy and echocardiogram results	
Check for renal and hepatic function	
Establish length of antifungal therapy	

### Intervention

The care bundle and quality “indicators” were extracted after having reviewed and collectively discussed the best practice advances in the management of candidemia [10]. The quality indicators were required to adhere to the following criteria: (1) the indicators are generally accepted clinical practice and supported by evidence; (2) the completion of each indicator can be determined by a yes or no on the checklist.

Six recommendations were therefore included in our care bundle for candidemia. Each one was provided in a structured form and checked on a list. Bundle endpoints included: (1) early (< 72 h) adequate antifungal therapy, (2) early (< 72 h) source control, if necessary, (3) follow-up blood cultures, (4) ophthalmologic examination, (5) echocardiogram and (6) adequate duration of therapy according to the complexity of the infection. Additionally, we also included a standardized checklist to gather bundle compliance data and to periodically check the clinical condition of the patients, microbiologic culture results and compliance with other measures that are generally accepted good clinical practice, such as drug selection and drug dosing according to hepatic and renal function, drug-drug interactions and drug de-escalations (Table 1).

The study was approved by the institutional review board of the Hospital General Universitario Gregorio Maranon (MICRO.HGUGM.2015-068) and was in accordance with the Declaration of Helsinki. Written informed consent was obtained from each participating patient.

### Endpoints

Considering that among patients with candidemia 30-day mortality is mainly related to the underlying disease of the patients [9], and assuming that an adequate management of candidemia could have an impact on 14-day related mortality, the main outcome of our study was 14-day all-cause mortality. As secondary outcome, we considered the adherence to all the quality indicators of the *Candida* bundle and 30-day mortality rate.

### Definitions

An episode of candidemia was defined as a patient that had at least one peripheral blood culture positive for *Candida* species. Sepsis, severe sepsis or septic shock were recorded on the day of candidemia [14]. Pitt’s bacteremia score was defined according to the standard international criteria [15].

As for the source of infection, an episode of candidemia was considered catheter-related if (1) the catheter tip culture was positive with the same *Candida* species, (2) there was evidence of exit site catheter exudate with the same *Candida* species or (3) the differential time to positivity of BCs obtained from the catheter and peripheral veins was ≥ 2 h [16].

The urinary tract was considered the portal of entry in patients with urologic predisposing conditions (i.e., manipulation or obstruction of the urinary tract) and evidence of urinary tract infection caused by the same species of *Candida*.

The abdomen was considered to be the origin of the candidemia when a patient had evidence of abdominal infection and (1) a positive culture from the intra-abdominal space was obtained during surgery or by needle aspiration and/or (2) no other apparent sources of candidemia were detected. When a source of candidemia could not be identified, candidemia was defined as “primary”.

Patients were considered to have *Candida* septic metastasis when an infection due to the same *Candida* species occurred in a site that was distant from the source of the candidemia. In cases in which no culture was available, the distant infection had to be temporally related with the fungemia and with no alternative cause explaining the clinical condition.

Infective endocarditis was diagnosed according to the Duke criteria [17]; ocular candidiasis was classified based on previous criteria [2, 5]; septic thrombophlebitis required the presence of venous thrombosis, confirmed by imaging techniques, in the setting of persistent candidemia [10].

Early antifungal treatment was defined as adequate if a recommended dose of an antifungal drug was administered within 72 h after candidemia onset, and it was found to be effective by in vitro susceptibility testing. Early adequate source control was defined as removal of the indwelling catheter or surgical drainage of deep infection within 72 h after the index blood sample had been drawn. For patients with candidemia without metastatic infection, duration of antifungal therapy was considered adequate when it lasted at least 14 days from the first negative blood cultures. For patients with candidemia with metastatic infection (i.e., ocular candidiasis, lung metastasis) and/or other complications (i.e., thrombophlebitis), duration of treatment was considered adequate when it reached at least 4–6 weeks or even longer in case of infective endocarditis.

### Data Collection

Data were prospectively recorded on a standardized case report form that included demographics, comorbidities, predisposing risk factors within the preceding 30 days, clinical severity according to the Pitt score, source of the infection, adherence to the *Candida* bundle, clinical management of the patients including antifungal choice, length of therapy and adequate source control of the infection and all-cause mortality.

### Statistical Analysis

Descriptive statistics were used to summarize the data. Quantitative variables are reported as median and interquartile range (IQR) and categorical variables as counts (%). The chi-square test or Fisher exact tests were used to compare the distribution of categorical variables, including the clinical characteristics of the pre- and post-intervention period and the association between individual risk factors and mortality rate. The *t*-test or Mann-Whitney test was used to compare quantitative variables. Statistical significance was set at *p* < 0.05. The Kaplan-Meier curve was constructed to show the relationship between the intervention strategy and 14-day survival. The statistical analyses were performed using Microsoft SPSS PC+, version 15.0 (SPSS, Chicago, IL, USA).

## Results

**Fig. 1:**
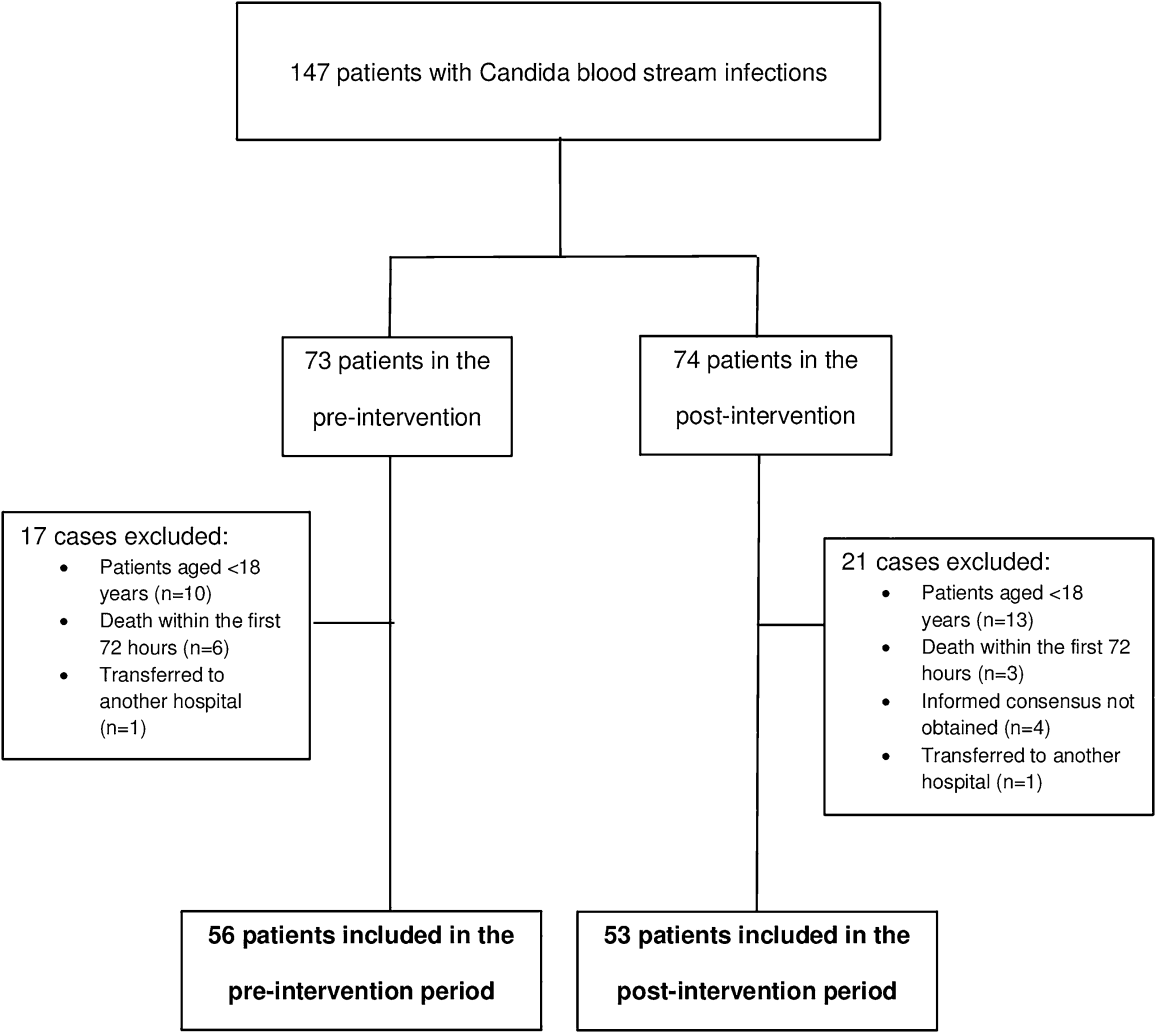
Flow chart of patients included in the study

The flowchart study is shown in Fig. 1. Overall, 147 patients were diagnosed with an episode of candidemia, 73 in the pre-intervention and 74 in the post-intervention period. In the pre-intervention period, ten patients were excluded because they were aged < 18 years, and six died within the first 72 h; one patient was transferred to another hospital. In the post-interventional period, 13 patients were excluded because they were aged < 18 years, 4 declined participation, 3 died within the first 72 h, and 1 was transferred to another healthcare facility.

The demographics and clinical features of the 109 patients in the study populations are shown in Table 2. Mean age was 67.2 years, and 73/109 patients were males (67.0%). The most common underlying condition was solid tumor [60/109 (55.0%)] followed by gastrointestinal disease [44/109 (40.7%)]. A central venous catheter (CVC) was in place in 86/109 patients (78.9%), and 70/109 (64.2%) were receiving total parenteral nutrition at the time of their episode. The most prevalent source of infection was the CVC [65/109 (59.6%)] followed by intra-abdominal origin [18/109; (16.5%)]. Only 13/109 patients (11.9%) had a primary infection.

**Table 2:** Comparison of patients with candidemia who were managed according to the comprehensive care bundle or not (controls)

Variables	Total population (*n* = 109)	Pre-intervention group (*n* = 56)	Post-intervention group (*n* = 53)	*p* value*
Age (years), mean ± SD	67.2 ± 13.9	66.6 ± 13.5	67.8 ± 14.4	0.67
Male, *n* (%)	73 (67.0)	37 (66.1)	36 (67.9)	1
Department, *n* (%)				
Medical ward	38 (34.9)	17 (30.4)	21 (39.6)	0.32
Surgical ward	36 (33.0)	23 (41.1)	13 (24.5)	0.07
ICU stay	23 (21.1)	10 (17.9)	13 (24.5)	0.48
Oncology-hematology ward	12 (11.0)	6 (10.7)	6 (11.3)	1
Underlying disease, *n* (%)				
Solid tumor	60 (55.0)	31 (55.4)	29 (54.7)	1
Gastrointestinal disease	44 (40.7)	25 (45.5)	19 (35.8)	0.33
Diabetes mellitus	26 (23.9)	12 (21.4)	14 (26.4)	0.65
Neurologic disease	26 (23.9)	12 (21.4)	14 (26.4)	0.65
Cardiovascular disease	25 (22.9)	13 (23.2)	12 (22.6)	1
Liver disease	15 (13.9)	10 (18.2)	5 (9.4)	0.26
Hematologic malignancy	6 (5.5)	2 (3.6)	4 (7.5)	0.43
Charlson comorbidity index, mean ± SD	3.6 ± 2.5	3.6 ± 2.6	3.6 ± 2.5	0.93
Risk factor, *n* (%)				
Central venous catheter	86 (78.9)	48 (85.7)	38 (71.7)	0.10
Total parenteral nutrition	70 (64.2)	35 (62.5)	35 (66.0)	0.84
Previous abdominal surgery	45 (41.3)	26 (46.4)	19 (35.8)	0.33
Corticosteroids	34 (31.2)	16 (28.6)	18 (34.0)	0.67
Previous antifungals	31 (28.4)	15 (26.8)	16 (30.2)	0.83
Neutropenia	8 (7.3)	5 (8.9)	3 (5.7)	0.71
Immunosuppressive therapy	8 (7.3)	3 (5.4)	5 (9.4)	0.48
Pitt score, median (IQR)	0 (0–2)	0 (0–2)	0 (0–2)	0.64
Time between hospitalization and candidemia onset, median (IQR)	23.0 (9.0–39.0)	22.0 (6.5–38.5)	23.0 (12.0–45.0)	0.55
Clinical manifestation, *n* (%)				
Sepsis	40 (36.7)	23 (41.1)	17 (32.1)	0.42
Severe sepsis	31 (28.4)	20 (35.7)	11 (20.8)	0.09
Septic shock	12 (11.0)	4 (7.1)	8 (15.1)	0.22
*Candida* species, *n* (%)				
*C. albicans*	56 (51.4)	25 (44.6)	31 (58.5)	0.18
*C. parapsilosis*	27 (24.8)	14 (25.0)	13 (24.5)	1
*C. glabrata*	15 (13.2)	8 (14.3)	7 (13.2)	1
*C. krusei*	6 (5.5)	4 (7.1)	2 (3.8)	0.67
*C. tropicalis*	4 (3.7)	2 (3.6)	2 (3.8)	1
Other *Candida* species^a^	3 (2.8)	3 (5.4)	0	0.24
Source, *n* (%)				
Central venous catheter	65 (59.6)	33 (58.9)	32 (60.4)	1
Intra-abdominal	18 (16.5)	12 (21.4)	6 (11.3)	0.20
Primary	13 (11.9)	6 (10.7)	7 (13.2)	0.77
Urinary tract	7 (6.4)	4 (7.1)	3 (5.7)	1
Others^b^	6 (5.5)	1 (1.8)	5 (9.4)	0.10
Initial antifungal therapy, *n* (%)				
Fluconazole	74 (67.9)	38 (67.8)	36 (67.9)	1
Echinocandins	29 (26.6)	15 (26.7)	14 (26.4)	1
Liposomal amphotericin B	6 (5.5)	3 (5.4)	3 (5.7)	1
ICU admission, *n (%)*	10 (9.2)	5 (8.9)	5 (9.4)	1
Length of hospital stay (days), median (IQR)	50.0 (31.0–88.5)	46.5 (22–86.7)	51 (37–97.5)	0.96

*ICU* intensive care units

^a^Other *Candida* species include: 1 *C. lusitaniae*, 1 *C. dublinensis* and 1 *C. incospicua*

^b^Other sources include: 1 chorioamnionitis, 2 peripheral catather; 2 infective endocarditis, 1 infection from prosthesis

*P values < 0.05 are shown in bold

### Comparison Between the Pre- and Post-intervention Period

The main demographic characteristic and risk factors for candidemia in the pre- and post-intervention period are compared in Table 2. Both populations were similar, and no statistically significant differences were detected regarding the demographics, underlying diseases, risk factors for candidemia, severity of disease and *Candida* species. However, in the pre-intervention period, patients had an intra-abdominal origin more often than those in the post-intervention period [12/56 (21.4%) vs. 6/53 (11.3%)], but this difference was not statistically significant (*p* = 0.20). During the pre-intervention period, ID physicians visited patients in 50/56 cases (89.2%), whereas 53/53 patients (100%) were visited in the post-intervention period (*p* = 0.71).

Overall, compliance with the *Candida* bundle significantly improved between pre- [27/56 (48.2%)] and post-intervention [43/53 (81.1%); *p* = 0.01] period (Table 3). Individual bundle components that significantly improved in the post-intervention period were early adequate antifungal therapy [47/56 (83.9%) vs. 51/53 (96.2%), *p* = 0.05], early adequate source control of the infection [37/56 (82.2%) vs. 41/53 (97.6%), *p* = 0.03] and appropriate duration of therapy [27/56 (48.2%) vs. 43/53 (81.1%), *p* = 0.01]. Moreover, adherence to follow-up blood cultures, ophthalmologic examination and echocardiography improved in the post-intervention period, but the difference was not statistically significant.

**Table 3:** Compliance with and impact of a comprehensive care bundle on candidemia

	Intervention group (*n* = 56)	Control group (*n* = 53)	*p* value*
All bundle elements successfully completed	27 (48.2)	43 (81.1)	**0.01**
Early adequate source control of infection	37 (82.2)	41 (97.6)	**0.03**
Early adequate antifungal therapy	47 (83.9)	51 (96.2)	**0.05**
At least one complication detected	10 (20.8)	19 (38.0)	0.08
Blood cultures every 48 h until negative	50 (89.3)	51 (96.2)	0.27
Persistent candidemia	15/51 (29.4)	8/51 (16.0)	0.15
Ophthalmologic examination performed	47 (83.9)	49 (92.5)	0.23
Ocular candidiasis	5/47 (10.6)	10/49 (20.4)	0.26
Echocardiograms performed	47 (83.9)	48 (90.6)	0.34
Trans-thoracic	22 (46.8)	18 (37.5)	
Trans-esophageal	25 (53.2)	30 (62.5)	
Infective endocarditis	0/46 (0)	2/48 (4.2)	0.49
Other complications			
Thrombophlebitis	4/9 (44.4)	5/11 (45.5)	1
Spread to other organs	1/56 (1.8)	7/53 (13.2)	0.03
Appropriate duration of therapy	45 (80.4)	51 (96.2)	**0.01**

*P values < 0.05 are shown in bold

### Analysis of All-cause Mortality at 14 and 30 Days

Overall, all-cause mortality at 14 and 30 days was 14.9% (16/109) and 27.5% (30/109). Variables associated with 14- and 30-day mortality in the univariate analyses are summarized in Tables 4 and Table 5. Being managed according to the candidemia bundle had a favorable impact on 14-day mortality [50/93 (53.8%) versus 3/16 (18.8%), *p* = 0.01] but not on the 30-day mortality rate [41/79 (51.9%) versus 12/30 (40%), *p* = 0.29]. However, after controlling for baseline characteristics, clinical presentation of candidemia and source of infection, being managed according to the candidemia bundle remained the only variable independently associated with a decreased all-cause mortality at both 14 (HR 0.08, 95% CI 0.01–0.45, *p* = 0.02) and 30 days (HR 0.40, 95% CI 0.18–0.89, *p* = 0.02) (Table 6).

**Table 4:** Univariate analysis of variables associated with 14-day mortality

Variables	Alive (*n* = 93)	Dead (*n* = 16)	*p* value*
Age (years), mean ± SD	66.3 ± 13.6	72.1 ± 15.3	0.13
Male, *n* (%)	62 (66.7)	11 (68.8)	1
Department, *n* (%)			
Surgical ward	34 (36.6)	2 (12.5)	0.08
Medical ward	29 (31.2)	9 (56.3)	0.08
ICU stay	21 (22.6)	2 (12.5)	0.51
Oncology-hematology ward	9 (9.7)	3 (18.8)	0.37
Underlying disease, *n* (%)			
Solid tumor	52 (55.9)	8 (50.0)	0.78
Gastrointestinal disease	38 (41.3)	6 (37.5)	1
Diabetes mellitus	23 (24.7)	3 (18.8)	0.75
Neurologic disease	23 (24.7)	3 (18.8)	0.75
Cardiovascular disease	19 (20.4)	6 (37.5)	0.19
Liver disease	13 (14.1)	2 (12.5)	1
Hematologic malignancy	4 (4.3)	2 (12.5)	0.21
Charlson comorbidity index	3.6 ± 2.6	3.4 ± 2.4	0.75
Risk factor, *n* (%)			
Central venous catheter	75 (80.6)	11 (68.8)	0.32
Total parenteral nutrition	62 (66.7)	8 (50.0)	0.26
Previous abdominal surgery	40 (43.0)	5 (31.3)	0.42
Corticosteroids	28 (30.1)	6 (37.5)	0.56
Previous antifungals	24 (25.8)	7 (43.8)	0.23
Neutropenia	6 (6.5)	2 (12.5)	0.33
Immunosuppressive therapy	7 (7.5)	1 (6.3)	1
Pitt score, median (IQR)	0 (0–2)	0 (0–1)	0.46
Clinical manifestation, *n* (%)			
Sepsis	35 (37.6)	5 (31.3)	0.78
Severe sepsis	28 (30.1)	3 (18.8)	0.54
Septic shock	8 (8.6)	4 (25.0)	0.07
*Candida* species, *n* (%)			
*C. albicans*	47 (50.5)	9 (56.3)	0.78
*C. parapsilosis*	24 (25.8)	3 (18.8)	0.75
*C. glabrata*	13 (14.0)	2 (12.5)	1
*C. krusei*	4 (4.3)	2 (12.5)	0.21
*C. tropicalis*	4 (4.3)	0	1
Other *Candida* species	3 (3.2)	0	1
Source, *n* (%)			
Central venous catheter	60 (64.5)	5 (31.3)	**0.02**
Intra-abdominal	14 (15.1)	4 (25.0)	0.29
Primary	8 (8.6)	5 (31.3)	**0.02**
Urinary tract	6 (6.5)	1 (6.3)	1
Other sources^b^	5 (5.4)	1 (6.3)	
Initial antifungal therapy, *n* (%)			
Fluconazole	63 (67.7)	11 (68.8)	1
Echinocandins	25 (26.8)	4 (25.0)	1
Liposomal amphotericin B	5 (5.3)	1 (6.3)	1
Early adequate antifungal therapy, *n* (%)	85 (91.4)	13 (81.3)	0.20
Early adequate source control of infection, *n* (%)	73 (93.6)	5 (55.6)	**0.006**
Persistent candidemia, *n* (%)	21 (23.1)	2 (20.0)	1
Ocular candidiasis, *n* (%)	15 (16.9)	0	0.59
Infective endocarditis, *n* (%)	2 (2.3)	0	1
ICU admission due to candidemia, *n* (%)	6 (6.5)	4 (25.0)	**0.04**
Intervention period, *n* (%)	50 (53.8)	3 (18.8)	**0.01**
All bundle elements successfully completed, *n* (%)	65 (69.9)	5 (31.3)	**0.004**

*ICU* intensive care units

^a^Other *Candida* species include: 1 *C. lusitaniae*, 1 *C. dublinensis* and 1 *C. incospicua*

^b^Other sources include: 1 chorioamnionitis, 2 peripheral catather; 2 infective endocarditis, 1 infection from prosthesis

*P values < 0.05 are shown in bold

**Table 5:** Univariate analysis of variables associated with 30-day mortality

Variables	Alive (*n* = 79, %)	Dead (*n* = 30, %)	*p* value*
Age (years), mean ± SD	66.5 ± 14.1	69.1 ± 13.7	0.40
Male, *n* (%)	26 (32.9)	10 (33.3)	1
Department, *n* (%)			
Surgical ward	33 (41.8)	3 (10.0)	**0.001**
Medical ward	25 (31.6)	13 (43.3)	0.26
ICU stay	14 (17.7)	9 (30.0)	0.19
Oncology-hematology ward	7 (8.9)	5 (16.7)	0.30
Underlying disease, *n* (%)			
Solid tumor	44 (55.7)	16 (53.3)	0.83
Gastrointestinal disease	35 (44.9)	9 (30.0)	0.19
Diabetes mellitus	21 (26.6)	5 (16.7)	0.32
Neurologic disease	20 (25.3)	6 (20.0)	0.62
Cardiovascular disease	16 (20.3)	9 (30.0)	0.31
Liver disease	12 (15.4)	3 (10.0)	0.55
Hematologic malignancy	2 (2.5)	4 (13.3)	**0.04**
Charlson comorbidity index, median (IQR)	3 (2–6)	3 (2–6)	0.53
Risk factor, *n* (%)			
Central venous catheter	62 (78.5)	24 (80.0)	1
Total parenteral nutrition	50 (63.3)	20 (66.7)	0.82
Previous abdominal surgery	36 (45.6)	9 (30.0)	0.19
Corticosteroids	21 (26.6)	13 (43.3)	0.10
Previous antifungals	20 (25.3)	11 (36.7)	0.24
Neutropenia	3 (3.8)	5 (16.7)	**0.03**
Immunosuppressive therapy	5 (6.3)	3 (10.0)	0.68
PITT score, median (IQR)	0 (0–2)	1 (0–3.25)	0.05
Clinical manifestation, *n* (%)			
Sepsis	32 (40.5)	8 (26.7)	0.26
Severe sepsis	24 (30.4)	7 (23.3)	0.63
Septic shock	4 (5.1)	8 (26.7)	**0.003**
*Candida* species, *n* (%)			
*C. albicans*	39 (49.4)	17 (56.7)	0.52
*C. parapsilosis*	19 (24.1)	8 (26.7)	0.80
*C. glabrata*	12 (15.2)	3 (10.0)	0.75
*C. tropicalis*	4 (5.1)	0	0.58
*C. krusei*	3 (3.8)	3 (10.0)	0.34
Other *Candida* species	3 (3.8)	0	0.56
Source, *n* (%)			
Central venous catheter	46 (58.2)	19 (63.3)	0.66
Intra-abdominal	14 (17.7)	4 (13.3)	0.77
Primary	8 (10.1)	5 (16.7)	0.34
Urinary tract	6 (7.6)	1 (3.3)	0.67
Other sources^b^	5 (6.3)	1 (3.3)	1
Initial antifungal therapy, *n* (%)			
Fluconazole	53 (67.1)	21 (70.0)	0.30
Echinocandins	23 (25.3)	6 (20.0)	0.34
Liposomal amphotericin B	3 (3.8)	3 (10.0)	0.28
Early adequate antifungal therapy, *n* (%)	71 (89.9)	27 (90.0)	1
Early adequate source control of infection, *n* (%)	61 (95.3)	17 (73.9)	**0.009**
Persistent candidemia, *n* (%)	16 (20.5)	7 (30.4)	0.39
ICU admission due to candidemia, *n* (%)	3 (3.8)	7 (23.3)	**0.004**
Intervention period, *n* (%)	41 (51.9)	12 (40.0)	0.29
All bundle elements successfully completed, *n* (%)	55 (69.6)	15 (50.0)	0.07

*ICU* intensive care units

^a^Other *Candida* species include: 1 *C. lusitaniae*, 1 *C. dublinensis* and 1 *C. incospicua*

^b^Other sources include: 1 chorioamnionitis, 2 peripheral catather; 2 infective endocarditis, 1 infection from prosthesis

*P values < 0.05 are shown in bold

**Table 6:** Cox regression analyses of variables associated with 14-day mortality among patients with *Candida* BSI

	14-Day mortality			30-Day mortality		
	HR	95% Confidence interval	*p* value*	HR	95% Confidence interval	*p* value*
Septic shock due to candidemia	**11.6**	**1.18–113.97**	**0.04**	2.04	0.62**–**6.73	0.24
Primary candidemia	**4.83**	**1.40–16.69**	**0.01**	2.82	0.94**–**8.44	0.06
ICU admission due to candidemia	3.92	0.90–16.21	0.06	**4.60**	**1.62–13.02**	**0.004**
Age ≥ 65 years	2.95	0.73–11.85	0.12	**2.6**	**1.10–6.33**	**0.03**
Male	1.47	0.40–5.38	0.55	1.57	0.63**–**3.92	0.33
Pitt score	0.62	0.32–1.17	0.14	1.00	0.80**–**1.24	0.98
Surgical ward	0.28	0.06–1.30	0.10	**0.14**	**0.04–0.51**	**0.003**
Intervention period	**0.08**	**0.01–0.45**	**0.004**	**0.40**	**0.18–0.89**	**0.02**

*P values < 0.05 are shown in bold

## Discussion

Our study shows that the combination of a comprehensive candidemia bundle with antifungal stewardship program involvement significantly improves adherence to guideline recommendations and overall management of patients with candidemia. This novel approach was effective and resulted in a reduction in 14- and 30-day all-cause mortality in the post-intervention group.

To our knowledge, this study is one of the largest and most inclusive study evaluating the clinical impact of a checklist care bundle for management of patients with candidemia. However, many published studies have described individual aspects of the bundle being associated with better prognosis (i.e., early adequate antifungal administration or adequate source control) [6–9]; only three recent studies have addressed the impact of a bundle approach on the management of patients with candidemia [18–20]. Antworth et al. [18] conducted a single-center study of 78 patients with candidemia and demonstrated improved compliance with all candidemia care bundle elements, but not significant differences in clinical outcome.

Takesue and colleagues [19] performed a nationwide study of 608 patients with candidemia to investigate compliance with their bundle and its impact on mortality. Unfortunately, study participation by infection control doctors was entirely voluntary, and the candidemia bundle was not systematically implemented in all candidemic patients. Reflecting this weakness, compliance with all bundle elements was particularly poor (6.9%), and the correlation between bundle compliance and either clinical success or mortality was not observed.

Finally, Gouliorius et al. implemented a similar study design to ours, but lacked the sample size to detect significant differences in mortality [12/44 (27%) versus 3/33 (9%), *p* = 0.08] [20].

In our study, the overall compliance with quality indicators was significantly improved in the intervention group, mainly driven by improvements in the administration of early adequate antifungal therapy, early source control of infection and adequate length of antifungal therapy. Notably, the improvements were demonstrated in a clinical setting with long experience in an antifungal stewardship program [12, 21, 22], in which the comparatively pre-intervention adherence to recommendations was particularly high (80.4–89.3%). These results highlight the importance of candidemia bundle implementation even in institutions with high baseline adherence and not only in hospitals with low baseline rates of bundle adherence, where the impact of the intervention could be even better than ours.

Regarding this aspect, the current study is the first demonstrating how the systematic implementation of a candidemia bundle can be associated with decreased all-cause mortality. Although previous studies showed that infectious disease consultation was associated with better management and patient outcome [11–13], we surprisingly found that 89.2% of the patients in the pre-intervention period were visited by an infectious disease specialist. We believe that using a structured “checklist” for making recommendations was crucial for reminding the infectious disease specialists about all the key aspects to consider for the management of patients with candidemia.

It is important to mention that other reasons could explain the better evolution of patients treated according to our candidemia bundle. First, our bundle was unique in that we not only looked at the intervention of implementing a candidemia protocol, but also looked at multiple aspects in the process of care, including management of other concomitant infections, medication toxicity and drug-drug interactions. All these items could have contributed to better use of antifungal therapy, especially in a population with multiple comorbidities such as ours. Second, in our bundle we included “adequate source control of the infection” rather than the “simple” CVC withdrawal included in all earlier studies as a major recommendation [18–20]. As previously reported, the benefit of early CVC withdrawal might be disputable when the source of candidemia is not the catheter [23, 24]. Considering that 30–40% of candidemic patients had an origin other than CVC, the more restrictive definition of adequate source control used by Antworth, Takesue and Goulorius [18–20] may explain why their reports were not able to find a significant association between the bundle interventions and mortality.

Finally, in contrast to previous studies, we did not include “de-escalation to fluconazole” [20] or “step-down therapy” [19] as a care element of our bundle. Although these components may play a significant role in terms of length of hospitalization and costs, its role in terms of improvement of patient outcome could be limited. In our opinion, future studies should be performed to compare candidemia bundles to identify one that is easier to perform and associated with a better outcome.

This study has some limitations that should be assessed. First, it is a quasi-experimental pre-post study design that lacks randomization. Thus, we may not have taken changes in standard of care for patients with candidemia during the study periods into account. Second, being a single-center study, the external validity should be confirmed. To note that our study was performed in a tertiary university hospital with a long history of antifungal stewardship and ID consultations, where all colleagues (even those in specialties other than infectious disease, e.g., surgeons, ophthalmologists, cardiologists) are clearly aware of the severity of *Candida* BSI. Therefore, the impact of the intervention could be not fully reproducible in other facilities without an antifungal stewardship program or ID consultation service. However, the results of this approach, based on the early involvement of an ID specialist in the management of patients with candidemia, could lead other healthcare organizations to implement collaborations with ID specialists. Third, our outcome measure was all-cause mortality, and we did not report any data on *Candida*-attributable mortality or mycologic response.

## Conclusion

In conclusion, according to our study hypothesis, the introduction of a comprehensive candidemia bundle with antifungal stewardship program involvement significantly improved adherence to quality indicators, overall management and clinical evolution of patients with candidemia. Our study encourages the systematic use of care bundles for the management of candidemia.
